# Effects of pharmacological delay with roxadustat on multi-territory perforator flap survival in rats

**DOI:** 10.1515/med-2023-0762

**Published:** 2023-07-31

**Authors:** Xianyao Tao, Xiaoyun Pan, Yongjun Rui, Mingyu Xue

**Affiliations:** Department of Hand Surgery, Wuxi 9th People’s Hospital Affiliated to Soochow University, Wuxi 214000, Jiangsu, China

**Keywords:** perforator flap, angiosomes, choke vessels, pharmacological delay, hypoxia-inducible factor, inducible nitric oxide synthase

## Abstract

Roxadustat (FG-4592) is a specific hypoxia-inducible factor (HIF) prolyl hydroxylase inhibitor. We investigated the effects of FG-4592 pretreatment on survival and second choke vessels of multi-territory perforator flaps in rats. In total, 72 rats were divided into two groups (*n* = 36 each): the experimental (FG-4592) group and the control group. FG-4592 was administered orally as a single dose of 60 mg/kg every other day; the first drug solution was administered to the animals 7 days before the surgical procedure. On postoperative day 7, the surviving flap area was calculated. At 12 h post-surgery, in the second choke zone in the flaps, macrovascular hinges were compared by angiography and imaging, and microvascular changes were assessed by histology. Laser Doppler imaging was used to evaluate flap perfusion at the second choke zone at 12 h and 7 days after surgery. At 7 days after surgery, the flap survival area and perfusion were significantly greater in rats given FG-4592 compared with controls. At 12 h after surgery, the diameter of macrovascular and microvascular vessels, nitric oxide content, perfusion, and the protein levels of HIF-1α and inducible nitric oxide synthase were also significantly greater in FG-4592-treated rats than controls. In conclusion, pretreatment with roxadustat may improve initial flap survival and dilate the second choke zone vessels in a multi-territory perforator flap.

## 1 Introduction

Large skin defects caused by trauma, burns, or cancer excision are typically repaired using multi-territory perforator flaps [1]. However, the prospective region that endures is frequently small and unstable. The number of angiosomes can be used to calculate the surviving area of a perforator flap, which is a new idea in flap design that has emerged in recent years as a result of angiosome theory. The anatomical, dynamic, and prospective territories are the three divisions of angiosomes in a flap [2]. Numerous investigations [3,4] have demonstrated that whereas the prospective area of a flap is susceptible to necrosis, the anatomical and dynamic territory is certain to survive. Large skin defects caused by trauma, burns, or cancer excision are typically repaired using multi-territory perforator flaps [1].

A choke vessel serves as a link between two adjacent angiosomes, A choke vessel – not an occluded one – is one whose caliber gradually decreases as a result of an anastomosis. It serves as the nearby angiosome’s primary blood supply [5,6,7]. Between the dynamic and prospective territory, a second choke zone exists [8]. Studies [9,10] have demonstrated a strong correlation between the second choke zone vessel’s size and the potential territory’s necrosis. In a multi-territory perforator flap, the second choke vessel is therefore crucial for the survival of the third territory.

The study of choke vessels is mostly derived from research about the surgical delay procedure [3,8]. As the methods of flap delay are improved, the pharmacological delay has become a focus of research because of its advantages in terms of safety, reduced trauma, and low pain level [11]. There have been few reports on the influence of pharmacological delay on the choke vessel.

Hypoxia-inducible factor-1 (HIF-1) is the master regulator of many processes that occur during adaptation to hypoxia. HIF-1 is a heterodimer composed of an oxygen-sensitive α-subunit (HIF-1α) and a constitutively expressed β-subunit (HIF-1β) [12]. Under normoxia, the HIF-1α subunit is hydroxylated by specific prolyl hydroxylases (PHD) and degraded rapidly via the ubiquitin–proteasomal system. In a hypoxic environment, the activity of PHD-mediated hydroxylation is inhibited, leading to the accumulation of HIF-1α in the nucleus, where it dimerizes with HIF-1β to drive the transcription of target genes. HIF-1α regulates the expression of more than 100 genes [13,14], including erythropoietin (EPO), inducible nitric oxide synthase (iNOS), etc., which play important biological roles [15,16].

Nitric oxide (NO) plays an important role in delayed flaps by dilating vessels and improving the microcirculation [17]. Our previous experiments have shown that NO derived from iNOS plays an important role in the second choke zone vasodilation and is important for the survival of a multi-territory perforator flap [18].

Our study investigated the effects of pharmacological delay with roxadustat (FG-4592) [19], a specific HIF PHD inhibitor, on the survival and choke vessels of multi-territory perforator flaps in a rat model, explored the possible mechanism underlying the effect of the target gene of HIF-1α, iNOS, on the survival and choke vessels of multi-territory perforator flaps in a rat model, and developed a novel method for harvesting large, extended perforator flaps.

## 2 Materials and methods

### 2.1 Animals and flap animal model

**Figure 1:**
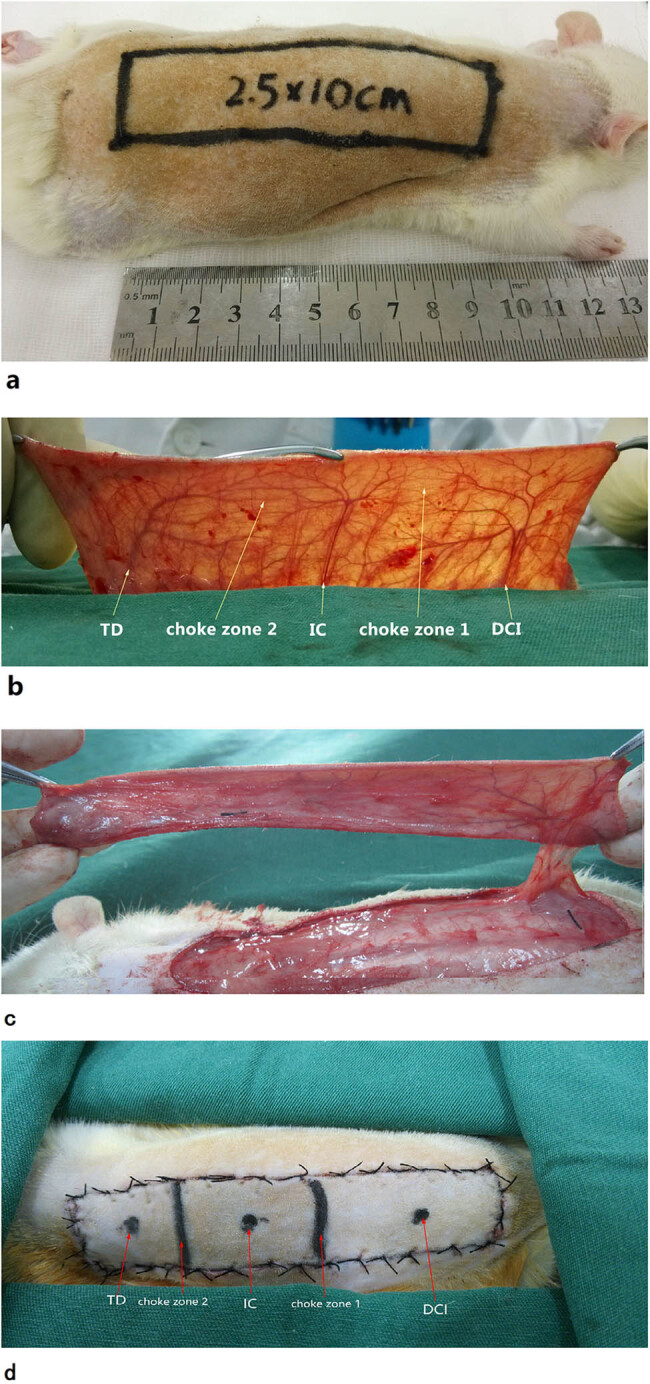
Flap outlining. (a) Design of the skin flap model. (b) Three vascular territories and two choke zones of the skin flap on the rat dorsum were observed, containing the thoracodorsal (TD), posterior intercostal (IC), and the deep circumflex iliac (DCI) vessels, and choke zones 1 and 2. (c) The flap was raised with pedicle. (d) The flap was sutured back into its original position; the three vascular territories and two choke zones were marked using a marker pen.

A total of 72 adult male Sprague–Dawley rats (weight: 250–300 g each) were obtained from the Experimental Animal Center, Soochow, China. The animals used in this study received humane care in compliance with the Guide for the Care and Use of Laboratory Animals. All animals were housed in solitary rat cages at an appropriate temperature (25°C) and were fed standard rat chow and tap water. Rats were anesthetized by intraperitoneal injection of 3% sodium pentobarbital (Merck & Co, NJ, USA) and sodium chloride solution (2 ml/kg). Additional doses (10–20%) were given as necessary during surgery. Dorsal hair was removed using an electric shaver, rats were fixed in the prone position on the operating table, and all surgical procedures were performed under sterile conditions. The entire dorsal island skin flap measuring 2.5 cm × 10 cm was designed based solely on the deep circumflex iliac artery of the right side (Figure 1a). This flap was reported by Miyamoto et al. [9] and contains three evenly sectioned vascular territories: the thoracodorsal vessel (TD), the posterior intercostal vessel (IC), and the deep circumflex iliac vessel (DCI), in addition to two choke zones (Figure 1b). The flap position was standardized using bony landmarks on the rat dorsum. The flap borders were as follows: medial border, the midline of the dorsum; lateral border, a site 2.5 cm lateral to the midline of the back; caudal border, a line joining the lateral and medial border at the anterior iliac spine; and cranial border, a line joining the medial and lateral borders at the apex of the axilla. The flap was elevated beneath the panniculus carnosus. Then, the DCI was separated, the TD and the IC were ligatured and cut off (Figure 1c), and the flap sutured back into its original position with 4-0 silk. If the vascular pattern was two or four angiosomes, the rat was excluded from the study. During the procedure, the positions of choke zones were observed carefully and were marked in the flap using a marker pen [20] (Figure 1d). The study was approved by the Wuxi 9th People’s Hospital Affiliated to Soochow University.

### 2.2 Administration of drugs

Animals were randomly divided into the following two groups of 36 animals each. Group A (the experimental (FG-4592) group) rats received oral FG-4592 (Selleck Chemicals, Houston, TX, USA) as a single dose of 60 mg/kg every other day and Group B (the control group) rats received only tap water during the experiment. The dose of FG-4592 used was based on a patent [19]. The first drug solution was administered to the animals 7 days before the surgical procedure, and FG-4592 was administered using a mouth gag and feeding tube. The final administration was performed 2 h before the surgical procedure.

### 2.3 Estimation of the survival area in the flap tissue

All flaps (six rats in each group) were observed for 7 days postoperatively. Rats were anesthetized and high-quality photographs of the dorsal skin flaps were obtained using a digital camera. The survival area of the flap was measured as a percentage of the total flap area, calculated using Image-Pro Plus, version 6.0 (Media Cybernetics, Rockville, MD, USA). The flap appearance, color, texture, and hair conditions were also evaluated [21]. Then, the rats were euthanized by administration of high doses of anesthetic.

### 2.4 Flap microangiography

Rats were anesthetized at 12 h postoperatively. Six Sprague–Dawley rats from each group underwent whole-body angiography according to a modified lead oxide-gelatin (Shanghai Chemical, Shanghai, China) injection technique using a 24-gauge intravenous silicone catheter. The right common carotid artery was injected with 1.5 ml of 1% heparin saline, followed by an injection of 150 ml/kg contrast medium (a mixture of lead oxide, gelatin, and water). After 24 h of fixation, the flaps were obtained, and radiographs were obtained (54 kV, 40 mA, 100 ms exposure) using a soft X-ray machine [22].

Then, using the electronic X-radiographs stored in the Picture Archiving and Communication Systems (PACS) (INFINITT Technology, Shanghai, China), we selected three larger vessels in the second choke zone of each flap and used the measurement tool included in PACS to measure the diameter of vessels. In total, 18 vessels were measured for each group.

### 2.5 Histology of microvessels

All tissue specimens (six rats per group) were obtained from the second choke zone of flaps as transverse sections and fixed in 4% paraformaldehyde at 12 h postoperatively. Paraffin-embedded samples were sectioned (5 μm thick) and stained with hematoxylin and eosin stain. The tissues were observed under a light microscope (100× magnification), and the 20 largest visible blood vessels were counted in 10 randomly chosen visual fields for each sample of the second choke zone after each slide identity was blinded. Vascular caliber was measured using DP2-BSW software (A4860300–35FEC5DC; Olympus Corporation, Tokyo, Japan). In total, 120 vessels in 60 randomly chosen visual fields were analyzed for each group.

### 2.6 Skin NO content assay

Six Sprague–Dawley rats of each group were anesthetized, and 0.2 g of skin samples of the second choke zone were weighed and homogenized in 1.8 ml of 0.9% saline. Then, the homogenates were centrifuged at 4,000 rpm for 5 min at 4°C, and the supernatant was subjected to NO assay and total protein determination. NO was assayed spectrophotometrically by measuring the total nitrate plus nitrite (NO^3−^ plus NO^2−^) and the stable end products of NO metabolism using the NO content assay kits (Nanjing Jiancheng Bioengineering Institute, Nanjing City, China). The total protein concentration was measured using the Coomassie blue method with bovine serum albumin as a standard [23]. In this procedure, nitrate is enzymatically converted into nitrite by the enzyme nitrate reductase, which is followed by quantitation of nitrite using the Griess reagent at an absorbance of 550 nm [24]. Results are expressed as μmol/g protein.

### 2.7 Western blot assay for HIF-1α and iNOS

For western blot assay, the total cellular protein was extracted from rat tissues derived from the second choke zone (six rats per group) using the radioimmunoprecipitation assay (RIPA) lysis buffer (Santa Cruz Biotechnology, Dallas, TX, USA) at 12 h postoperatively. The tissue samples were homogenized and centrifuged at 12,000 × *g* for 10 min at 4°C and the protein concentration of the supernatant was measured using the commercially available Bradford reagent (Sigma-Aldrich, St Louis, MO, USA). Protein was separated in 8% sodium dodecyl sulfate–polyacrylamide gels (Sigma-Aldrich) and transferred to polyvinylidene fluoride membranes (Roche Applied Science, Indianapolis, IN, USA). The membranes were blocked for 2 h at room temperature with 5% nonfat dry milk in buffer. Then, they were separately incubated at 4°C overnight with 1:800 diluted specific rabbit anti-rat iNOS antibody (Abcam, Cambridge, MA, USA), 1:200 diluted specific rabbit anti-rat HIF-1α antibody (Abcam, Cambridge, MA, USA), or 1:1,000 diluted specific rabbit anti-rat GAPDH antibody as an internal control (Abcam, Cambridge, MA, USA) and probed with the respective secondary antibodies (Rockland Immunochemical, Inc., Gilbertsville, PA, USA), for 2 h at room temperature. Bands were then detected using enhanced chemiluminescence (ECL) plus reagent (Thermo Fisher Scientific, Rockford, IL, USA) by ECL detection (Perkin Elmer, Waltham, MA, USA). Band intensity was quantified using AlphaEaseFC 4.0 software and presented relative to GAPDH. This experiment was performed in triplicate.

### 2.8 Flap perfusion laser Doppler imaging

Flap perfusion was measured using a laser Doppler perfusion imager (Lisca PIMII, Stockholm, Sweden). The imager has a probe for both plane curve (imaging mode) and continuous recording (perfusion monitor mode). The rats (six rats per group) were maintained at 24°C and anesthetized at 12 h and 7 days postoperatively. Flap perfusion imaging was done using a blood perfusion monitor (PeriFlux System 5000; PERIMED, Järfälla, Sweden) and it began 60 min after stabilization. The second choke zone was inspected for 1 min. The mean blood flow was expressed as perfusion units [25].

### 2.9 Statistical analyses

SPSS software version 19.0 (SPSS, Chicago, IL, USA) was used for statistical analyses. Normally distributed data are expressed as mean ± SD. The two groups were compared using the independent Student’s *t*-test and one-way repeated measures analysis of variance. Statistical significance was accepted at *P* < 0.05.


**Ethics approval and consent to participate:** The study was approved by the Wuxi 9th People’s Hospital Affiliated to Soochow University.

## 3 Results

### 3.1 Flap survival area

**Figure 2:**
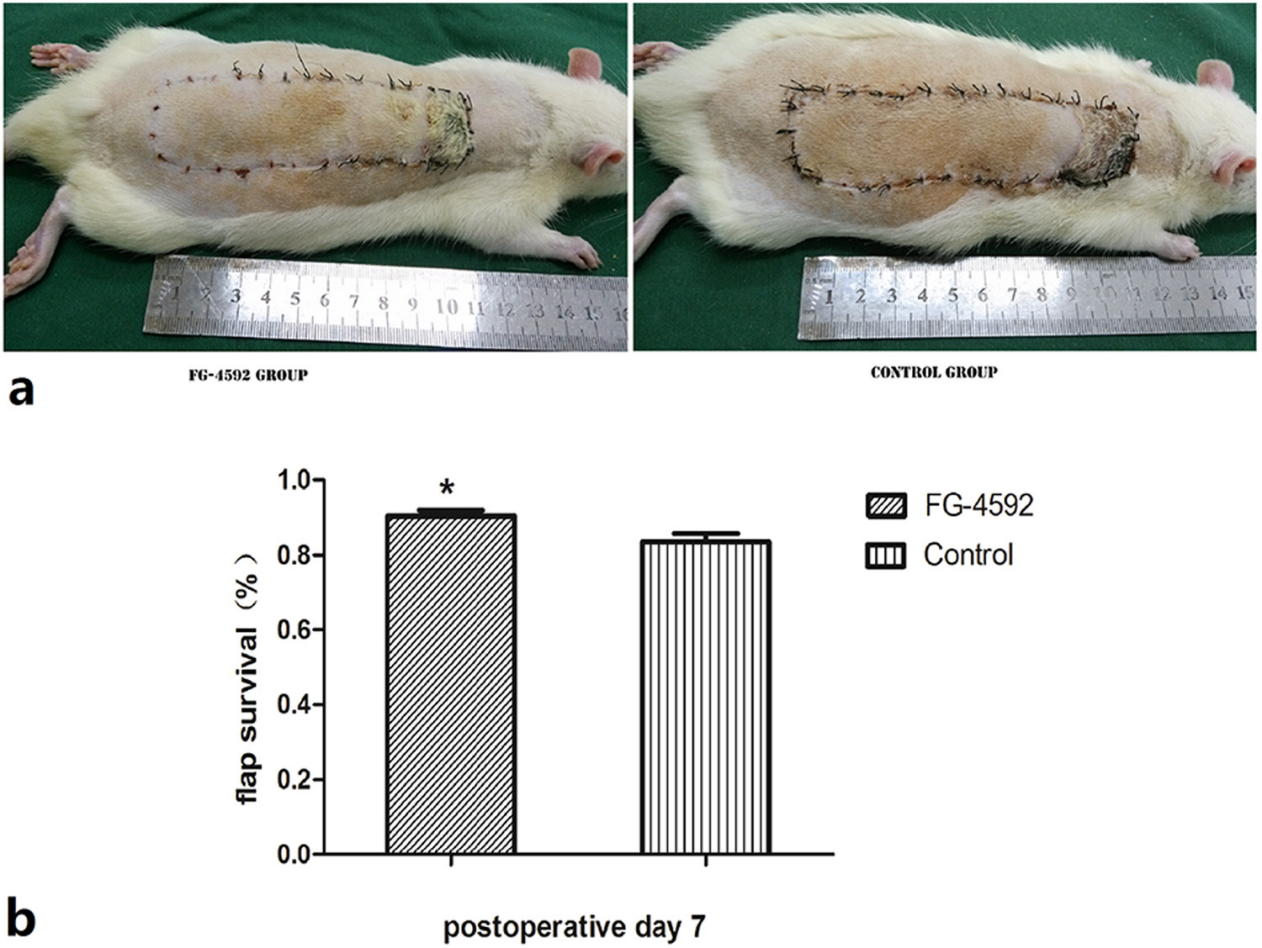
Flap survival. (a) Digital photographs indicating survival of the flaps from rats treated with FG-4592, and control group on postoperative day 7. (b) Postoperative day 7, the flap survival area relative to the total flap area (per cent) of the FG-4592 group shown as mean ± SD. *n* = 6 animals/group. *P < 0.05 compared with the control group.

No animals died from the drug treatment or the surgical procedure. At 12 h after surgery, the flaps in the two groups swelled and became dark purple to some extent. At day 7, regions of survival and necrosis were clearly demarcated in the two groups; the necrotic component tended to fuse, scab, and shrink (Figure 2a). The mean flap survival area in the FG-4592 group was significantly higher than that in the control group (89.40 ± 4.14% vs 82.61 ± 2.77%; *P* < 0.05; Figure 2b). Flap necrosis occurred only in the third territory in the two groups.

### 3.2 Vessel diameter in histology and angiography

**Figure 3:**
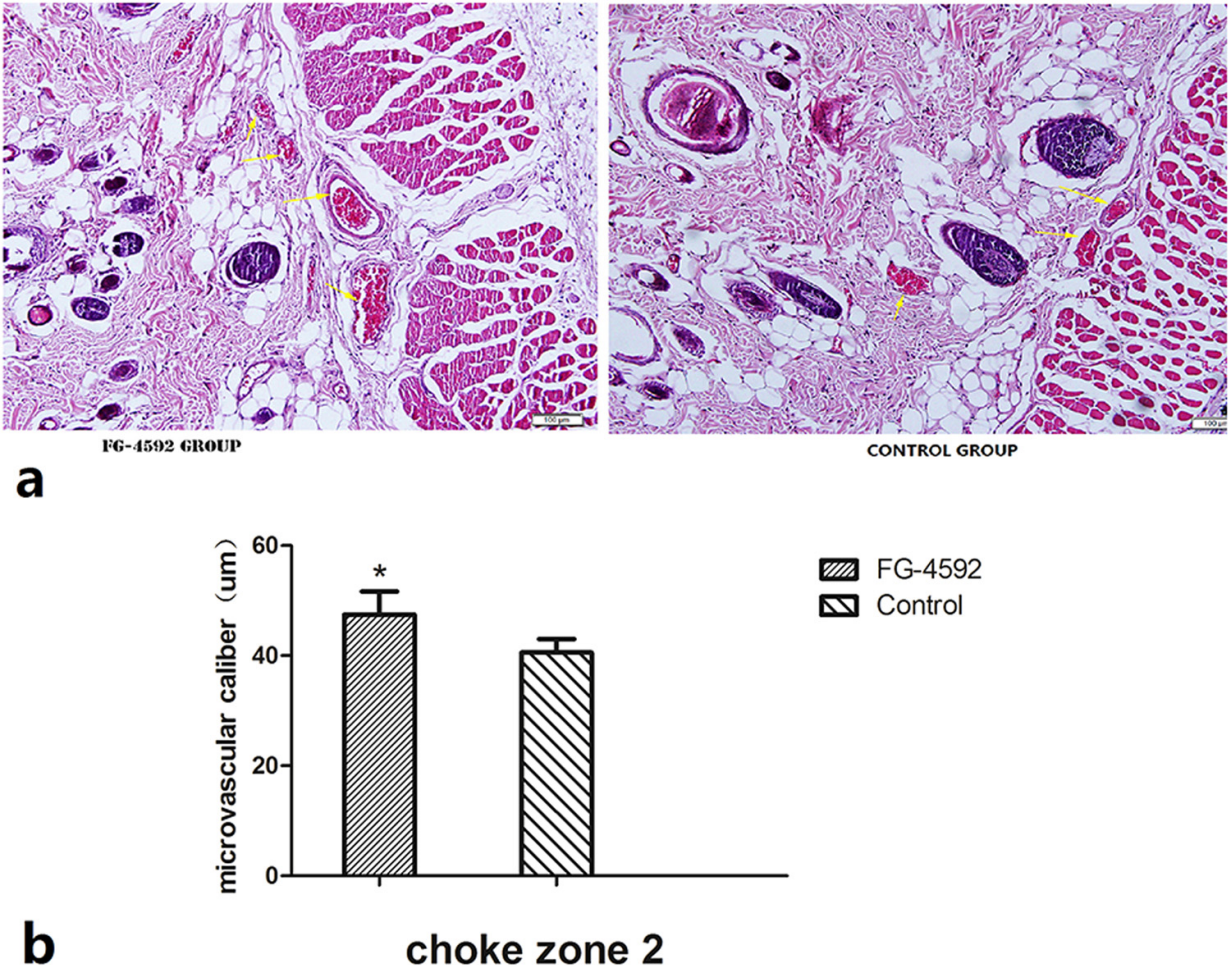
The diameter of microvascular vessels by histology. (a) Axial dorsal flaps were excised, fixed, and stained with hematoxylin and eosin. Tissue specimens in the second choke zone at 12 h postoperatively were then examined under ×100 magnification; yellow arrows indicate vessels containing red blood cells. (b) The mean vessel diameter of the second choke zone in the FG-4592 group was significantly larger than that in controls, *n* = 6 animals/group. *P < 0.05.

**Figure 4:**
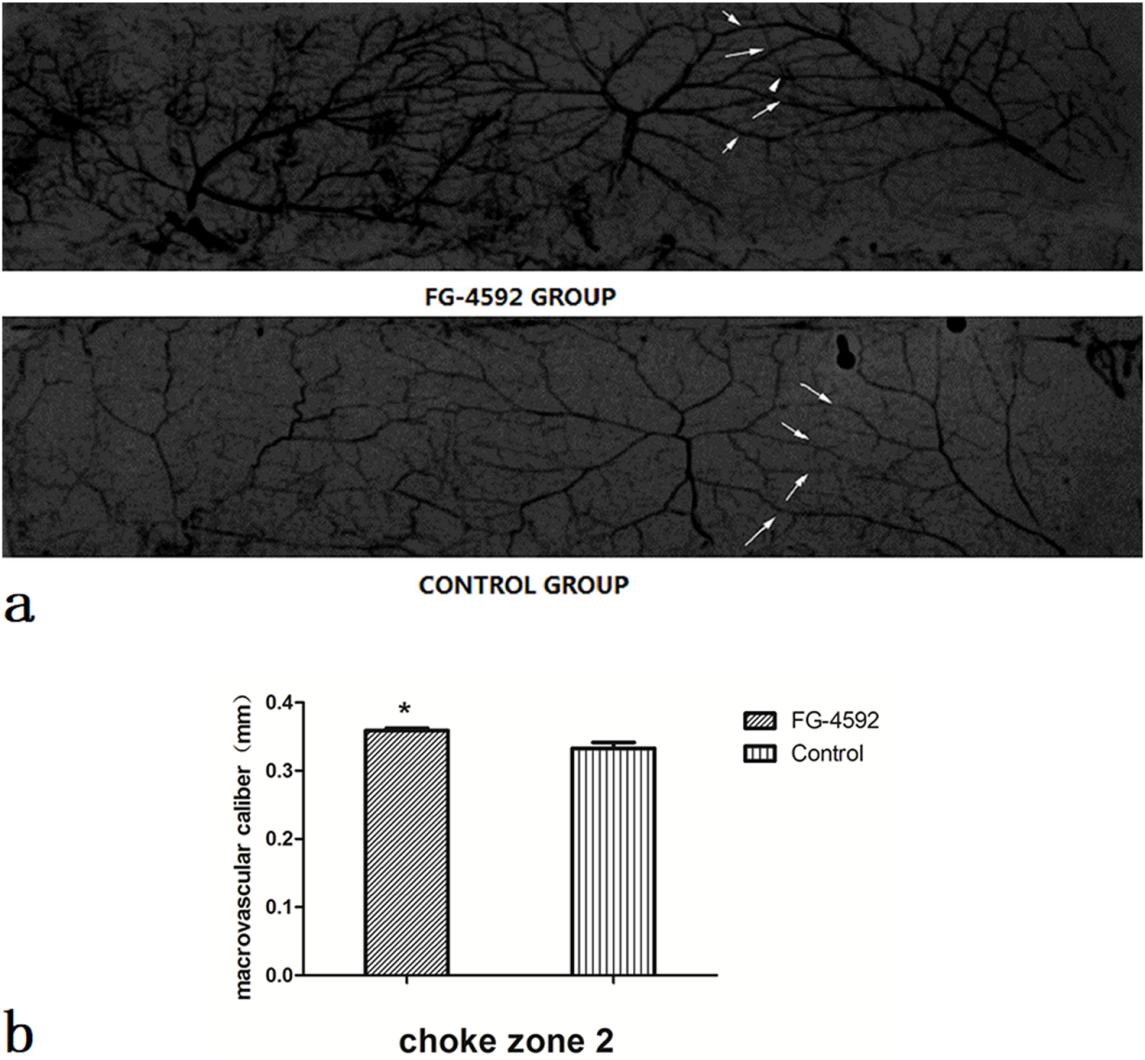
The diameter of macrovascular vessels by angiography. (a) X-ray picture from the PACS. We used the PACS measurement tool to measure the caliber of the three larger vessels in each flap, and representative arteriograms of rats from the four groups injected with a lead oxide and gelatin compound are shown. The right side of each flap is the cranial side, and the upper side of each is the medial side. White arrows indicate vessels in the second choke zone. (b) The mean vessel diameter of the second choke zone in the FG-4592 group was significantly larger than that in controls, *n* = 6 animals/group. *P < 0.05.

The diameters of macrovascular and microvascular vessels in the second choke zones were assessed using arteriography and histology at 12 h post-surgery (Figures 3a and 4a). On histology, the mean vessel diameter of the second choke zone in the FG-4592 group was significantly larger than that in controls (47.50 ± 4.17 vs 34.35 ± 2.14 µm; *P* < 0.05; Figure 3b). On angiography, the mean vessel diameter of the second choke zone in the FG-4592 group was significantly larger than that in controls (0.353 ± 0.022 vs 0.329 ± 0.015 mm; *P* < 0.05; Figure 4b).

### 3.3 Protein levels of HIF-1α and iNOS

**Figure 5:**
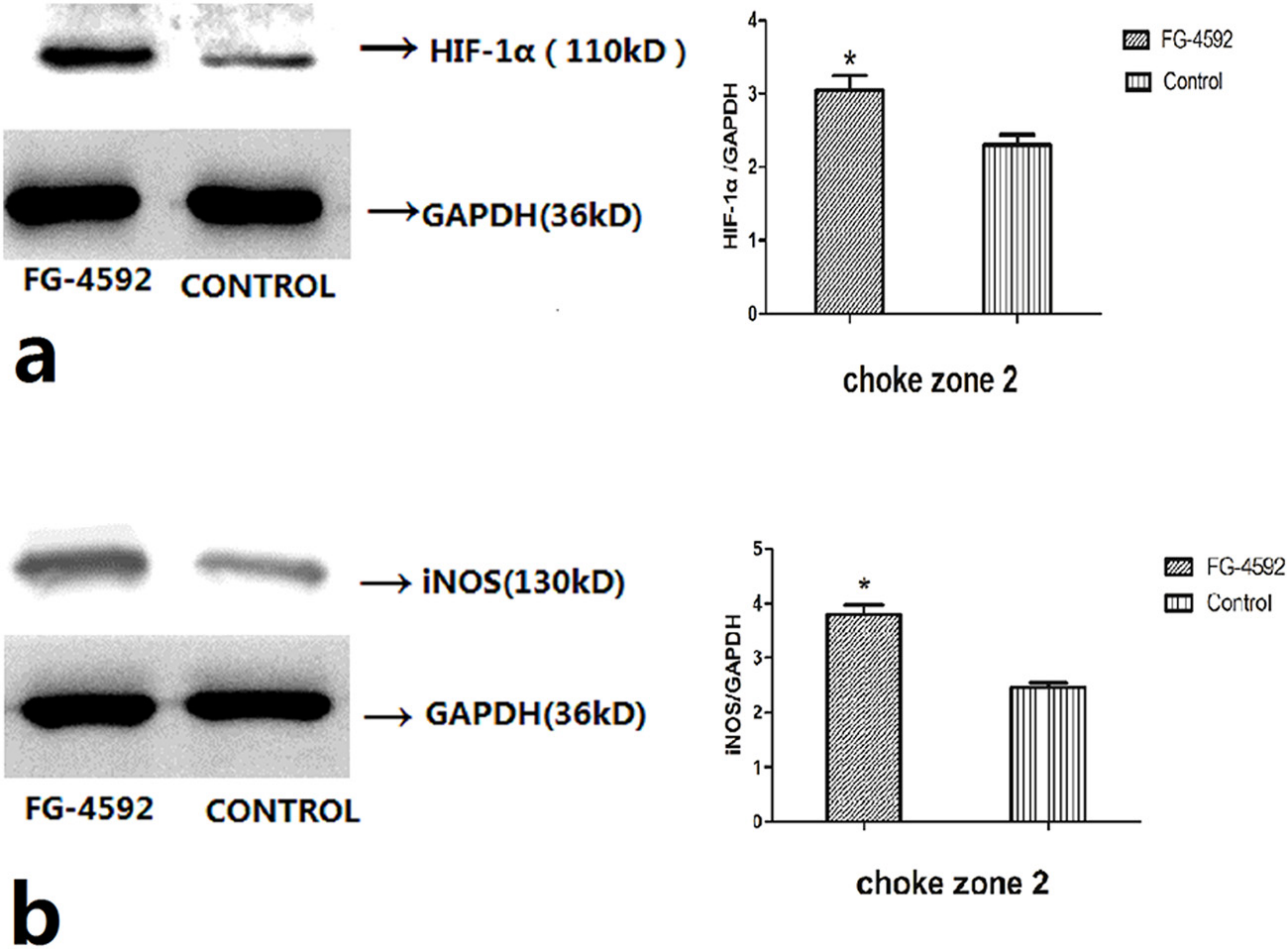
HIF-1α and iNOS expression. (a) FG-4592 promotes stabilization and activation of HIF-1α by Western blotting in the second choke zone at 12 h post-surgery. GAPDH served as a loading control. The protein levels of HIF-1α was calculated relative to that of GAPDH. *n* = 6 animals/group. *P < 0.05 compared with the control group. (b) FG-4592 promotes expression of iNOS by Western blotting in the second choke zone at 12 h post-surgery. GAPDH served as a loading control. The protein levels of iNOS was calculated relative to that of GAPDH. *n* = 6 animals/group. *P < 0.05 compared with the control group.

To evaluate the ability of FG-4592 to stabilize HIF-1α protein, we determined the protein level of HIF-1α in the second choke zone of flaps treated with FG-4592. Western blot analysis showed that FG-4592 treatment increased the protein level of HIF-1α significantly compared with the control group (*P* < 0.05; Figure 5a). iNOS is one of the target genes of HIF-1α and has been implicated in flap survival although its role remains controversial. Thus, we focused on iNOS and examined its expression in the second choke zone of the flap. The expression of iNOS was significantly higher in FG-4592-treated rats compared with the control group (*P* < 0.05; Figure 5b).

### 3.4 NO content

**Figure 6:**
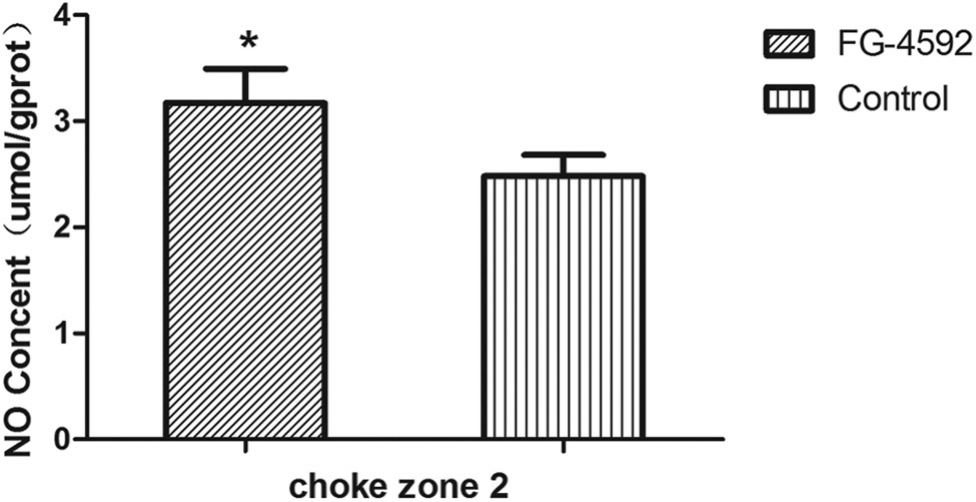
The choke zone of NO content. Mean (SD) NO content of the second choke zone in the flaps in the two groups measured using NO assay kits at 12 h postoperatively. *n* = 6 animals/group. *P < 0.05 compared with the control group.

The NO content in the second choke zone was measured 12 h after surgery. The NO content in rats treated with FG-4592 were higher compared with control rats (3.17 ± 0.32 vs 2.48 ± 0.20 µmol/g protein; *P* < 0.05; Figure 6).

### 3.5 Blood flow

**Figure 7:**
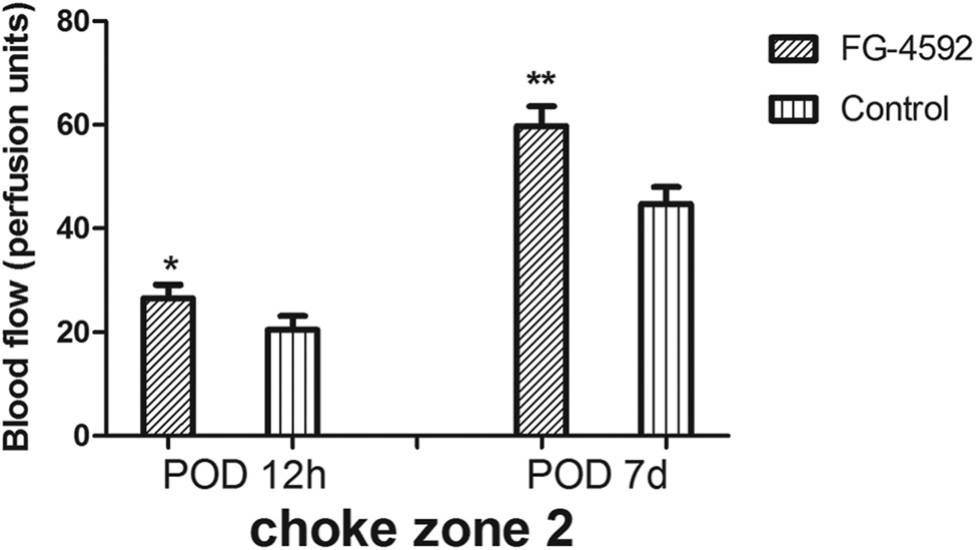
Blood flow. Mean (SD) blood flow of the second choke zone in the flaps in the two groups measured by laser Doppler imaging at 12 h after surgery and on postoperative day 7. *n* = 6 animals/group. *P < 0.05, **P < 0.05 compared with the control group.

The blood flow in the second choke zone was measured at 12 h and 7 days after surgery. At 12 h and 7 days after surgery, the same pattern of differences was observed. At 12 h after surgery, the mean blood flow of the second choke zone in rats treated with FG-4592 was higher compared with control rats (26.59 ± 2.58 vs 20.48 ± 2.71 perfusion unit; *P* < 0.05). At 7 days after surgery, the mean blood flow of the second choke zone in rats treated with FG-4592 was higher compared with control rats (59.76 ± 3.80 vs 44.68 ± 3.32 perfusion unit; *P* < 0.05) (Figure 7).

## 4 Discussion

According to Kerrigan and Daniel [26], the distal flap’s survival depends on adequate blood flow during the first 12 h following flap elevation. The second choke vessel is necessary for a multi-territory perforator flap to survive. The second choking zone must be dilated during the first 12 h if a substantial multi-territory perforator flap needs to be raised to suit the patient’s clinical needs. Pharmacological delay [27] is a non-surgical delay technique that employs medications rather than surgery to intervene and delay the flap to develop an adaptive response to support survival when the flap is exposed to ischemia. In the delayed flaps’ microcirculation, none was crucial [17].

The main regulator for adjusting to hypoxia is HIF-1. More than 100 genes, including EPO, iNOS, vascular endothelial growth factor (VEGF), and heme oxygenase-1, are regulated by HIF-1 [13,14]. These genes are involved in a range of responses to hypoxia, including angiogenesis, erythropoiesis, cell survival, proliferation, and differentiation [15,16,28]. However, it is yet unknown how the NO produced by iNOS, which has its origins in HIF-1 expression, affects flap delay.

Roxadustat (FG-4592) [19] is an oral HIF PHI inhibitor that mimics the body’s response to high-altitude hypoxic regions, inducing activation of a series of target genes, including EPO, EPO receptor, proteins promoting iron absorption, iron transport, and heme synthesis [29], and it is a stable hypoxia-inducing factor. Whether it is for patients with chronic kidney disease who have not received dialysis, or for patients with end stage renal disease who require dialysis, FG-4592 can be used to treat and maintain certain hemoglobin levels in anemia patients. Clinical trials are ongoing [30]. In our study, we used FG-4592 as a hypoxia-mimetic drug to prolong the stability and activity of HIF-1α in the second choke zone. Western blotting showed that the iNOS level was increased significantly after pre-treatment with FG-4592, which paralleled the HIF-1α activation, showing that FG-4592 stabilizes HIF-1 α and improves the biological activity of iNOS. We examined the effects of FG-4592, which enhances the expression of iNOS and increases the NO levels, on the second choke zone in rats, and we found that the level of iNOS corresponded to the NO level.

The flap survival area in rats treated with FG-4592 was higher than that in the control group. The diameter of vessels in the second choke zone of rats treated with FG-4592 was larger than that in the control group, based on angiography and histological evaluations. The laser Doppler observations demonstrated that the blood flow in the second choke zone was maintained at relatively higher levels in the FG-4592 group than in the control group. The blood flow and vessel diameter results suggest that vascularization was increased in the second choke zone, probably via the vasodilator effect of NO, which was regulated by the HIF-1α-enhanced expression of iNOS. This early enhancement in the diameters of second choke vessels should lead to increased survival of multi-territory perforator flaps. In our experiment, inducing flap delay by preconditioning with the PHD inhibitor FG-4592 improved the survival of multi-territory perforator flaps. The probable mechanism is NO derived from iNOS dilating the second choke vessel in the early period via HIF-1α activation. We also found that the enhanced HIF-1α promoted the expression of VEGF, which had an angiogenic effect and improved the flap survival. In our study, we could not demonstrate the effect of VEGF. Therefore, further experiments are needed to confirm the role of VEGF in flap survival.

In conclusion, we believe that pharmacological delay with FG-4592 efficiently increases the expression of NO generated from iNOS, thereby hence enhancing the vasodilatation of choke arteries, based on the experimental data and flap survival. This markedly boosted postoperative blood perfusion of an ischemic extended flap and flap survival. This is a cutting-edge method to lessen the necrotic region in the prospective territory of a multi-territory perforator flap and should help patients with significant wound defects brought on by burns, trauma, or tumor excision, particularly in anemic patients.

## Abbreviations

NO: nitric oxide
PHD: prolyl hydroxylase
EPO: erythropoietin
iNOS: inducible nitric oxide synthase
TD: thoracodorsal vessel
IC: intercostal vessel
DCI: deep circumflex iliac vessel
PACS: Picture Archiving and Communication Systems
